# An improved method for functional similarity analysis of genes based on Gene Ontology

**DOI:** 10.1186/s12918-016-0359-z

**Published:** 2016-12-23

**Authors:** Zhen Tian, Chunyu Wang, Maozu Guo, Xiaoyan Liu, Zhixia Teng

**Affiliations:** 1 Department of computer Science and Engineering, Harbin Institute of Technology, Harbin, 150001 People’s Republic of China; 2 Department of Information Management and Information System, Northeast Forestry University, Harbin, 150001 People’s Republic of China

**Keywords:** Gene Ontology, Specificity of terms, Weighted inherited semantics, Gene functional similarity

## Abstract

**Background:**

Measures of gene functional similarity are essential tools for gene clustering, gene function prediction, evaluation of protein-protein interaction, disease gene prioritization and other applications. In recent years, many gene functional similarity methods have been proposed based on the semantic similarity of GO terms. However, these leading approaches may make errorprone judgments especially when they measure the specificity of GO terms as well as the IC of a term set. Therefore, how to estimate the gene functional similarity reliably is still a challenging problem.

**Results:**

We propose WIS, an effective method to measure the gene functional similarity. First of all, WIS computes the IC of a term by employing its depth, the number of its ancestors as well as the topology of its descendants in the GO graph. Secondly, WIS calculates the IC of a term set by means of considering the weighted inherited semantics of terms. Finally, WIS estimates the gene functional similarity based on the IC overlap ratio of term sets. WIS is superior to some other representative measures on the experiments of functional classification of genes in a biological pathway, collaborative evaluation of GO-based semantic similarity measures, protein-protein interaction prediction and correlation with gene expression. Further analysis suggests that WIS takes fully into account the specificity of terms and the weighted inherited semantics of terms between GO terms.

**Conclusions:**

The proposed WIS method is an effective and reliable way to compare gene function. The web service of WIS is freely available at http://nclab.hit.edu.cn/WIS/.

**Electronic supplementary material:**

The online version of this article (doi:10.1186/s12918-016-0359-z) contains supplementary material, which is available to authorized users.

## Background

Gene Ontology (GO) is a standardized, precisely defined and controlled vocabulary of terms. It comprises three orthogonal ontologies: *cellular component* (CC), *molecular function* (MF) and *biological process* (BP) [1]. These ontologies are structured as three directed acyclic graphs (DAGs) in which, the nodes correspond to the terms describing a certain biological semantic category and the edges represent the linkages between terms describing defined relationships [2]. Genes and gene products in many biomedical databases such as UniProt [3], SwissProt [4] have been annotated by GO terms [5, 6]. Therefore, semantic similarity applied to GO annotations of genes can provide a measure of their functional similarity.

In recent years, many gene functional similarity methods based on GO [2, 5, 7–19] have been proposed by researchers. These measures have been widely used in all kinds of important applications such as protein-protein interaction prediction [20–23], network prediction [24–26], cellular localization prediction [27], disease gene prioritization [8, 28, 29], pathway modeling [30] and improving analysis of microarray data quality [31]. Measuring the functional similarity is more informative for understanding the biological roles and functions of genes, although sometimes it may be less objective and striking comparing with sequence and structure similarity [5, 32, 33].

Information content (IC) is an important dimension of word knowledge since it gives a measure how specific a term is [34]. There are mainly two approaches named corpus-based and structured-based, which could measure the IC of terms. The IC of a term *t* based on the corpus-based approach is defined as

1 $$ IC(t)=- \log \left(p(t)\right) $$

where *p*(*t*) is the probability of term *t* and its descendants occur in a certain corpus such as GOA database [14]. According to Eq. (1), the specificity of a term is fully dependent on the number of genes it annotates in a certain corpus. However, corpus-based approach is not reasonable enough for the definition of term IC since it may change with the daily evolution of GOA database [35].

Alternatively, IC of terms can also be computed based on GO graph. Firstly, Nuno [34] elaborated that terms with more descendants conveyed less information than terms that with fewer descendants. Therefore, the IC value of a term *t* can be formulated as:

2 $$ IC(t)=1-\left(\frac{ \log \left(des(t)\right)+1}{ \log \left( total\_ terms\right)}\right) $$

where des(*t*) denotes the descendants of term *t* and *total_terms* presents the total number of terms in the corresponding ontology. However, Teng et al. [32] further demonstrate that IC of terms is not only proportional to their depth but also inversely to the number of their descendants. Therefore, the IC of a term is achieved by

3 $$ IC(t)= depth(t)\times \left(1-\left(\frac{ \log \left(des(t)\right)+1}{ \log \left( total\_ terms\right)}\right)\right) $$

The estimation of semantic similarity between concepts is also an important component of analyzing natural language resources [36]. Afterwards, Sanchez [37] proposed a novel model for measuring IC of terms that both considered their ancestors and leaves. Sanchez’s model is designed as

4 $$ IC(t)=- \log \left(\frac{\frac{\left| leaves(t)\right|}{\left|AS(t)\right|}+1}{ \max \_ leaves+1}\right) $$

where AS(*t*) denotes the ancestor set of term *t* and max_leaves represents the total number of leaves in the ontology. Besides, leaves (*t*) is defined as

$$ leaves(t)=\left\{\left.l\in S\right|l\in hyponyms(t)\wedge l\  is\ a\  leaf\right\} $$

where *l* is a leaf iff *hyponyms*(*l*) = ∅ and S is the term set of the ontology. However, Sanchez’s model ignores the edge density and graph topology information in the different portions of the GO graph. At the same time, it also doesn’t consider the descendants of terms that are not leaves.

Measures of gene functional similarity can mainly be divided into two categories: pairwise approaches and groupwise approaches, both of which have to rely on the GO graph [31]. Pairwise methods measure gene functional similarity through two steps [32]. The first step is measuring semantic similarity scores of term pairs using term comparison techniques. The second step is to integrate semantic similarities of term pairs into a single functional similarity. Three distinct approaches which are average rule, maximum rule and best match average rule (BMA) have been proposed for the integration in the second step [38]. It is well accepted that the BMA rule is best overall. Pairwise approaches measure the semantic similarity between GO terms can be divided three categories: node-based, edge-based and hybrid [39].

Node-based measures [9–11, 40] are original developed for WordNet, and then applied to GO. Resnik [11] considered the most informative common ancestors (MICA) of two terms. Jiang and Conrath (JC) [9] and Lin [10] take into consideration the specificity of terms themselves, as well as the specificity of the most MICA. GraSM [40] considers average IC of all disjoint common ancestors rather than MICA only. However, these methods all suffer from ‘shallow annotation’ problem in which the semantic similarity values between terms near the root of the ontology are sometimes measured very high [5, 41].

Edge-based approaches [16, 19, 42, 43] calculate the number of edges along the paths that link two GO terms. The drawback of these approaches is that they assume all the edge in GO graph represents uniform distance and only count the number of edges on the paths traversed from one term to another. More recently, several researchers have attempted to address this issue by assigning different weights to edges that belong to different levels [15, 17]. However, they still ignore two important facts. One is the semantic similarity of two terms with a certain graph distance near the root would be equal to the semantic similarity of two terms with the same graph distance but away from the root. The other is that it is difficult to confirm weights of edges since the complex relationships of terms in the GO graph.

The hybrid methods [2, 12, 44, 45] not only consider the structure of the ontology but also distinguish the edges based on their different types and levels. Wang [12] designed a method that each edge is assigned a fixed weight according to the type of relationship between terms. The weight is also called semantics contribution factor (ω_e_). There are two mainly disadvantages of Wang’s method. One is that the semantic contribution factor (ω_e_) is fixed according the linking types of GO terms. The other is that the semantic contribution only depends on the maximum products of all the paths linking the two terms.

Groupwise methods measure gene functional similarity via comparing the terms that annotate genes in groups. According to Pesquita [39], there are three types of categories to measure the functional similarity of genes: set, graph and vector. Purely set-based approaches are not common, because few measures only consider direct annotations.

Many graph-based approaches [46–48] use set similarity techniques to simplify the problem of graph matching. These methods put the terms and their ancestors into a term set first. Then, they compute semantic similarity score between the term sets using Tversky’s ratio model [47]. Finally, the semantic similarity score between term sets is regarded as the gene functional similarity. Gentleman et al. [18] raised a method called simUI. It measures the similarity as the number of GO terms shared by two genes divided by the number of GO terms they have together. The functional similarity between *g*_1_ and *g*_2_ is:

5 $$ simUI\left({g}_1,{g}_2\right)=\frac{\left|{A}_{g1}\cap {A}_{g2}\right|}{\left|{A}_{g1}\cup {A}_{g2}\right|} $$

where *A*_*g*1_ and *A*_*g*2_ denote the term sets that annotate gene *g*_1_ and *g*_2_ respectively. According to [49], simGIC is an expansion of simUI that sums the IC of annotation terms. For two genes *g*_1_ and *g*_2_, simGIC is given by

6 $$ simGIC\left({g}_1,{g}_2\right)=\frac{{\displaystyle {\sum}_{t_i\in {A}_{g1}\cap {A}_{g2}}IC\left({t}_i\right)}}{{\displaystyle {\sum}_{t_j\in {A}_{g1}\cup {A}_{g2}}IC\left({t}_j\right)}} $$

While simUI does not consider the specificity of the terms in the GO graph, simGIC takes the IC of a term as its specificity. As is pointed out by Teng [32], simGIC ignores the shared IC between terms and this may also result in misjudgments for gene functional similarity.

Teng et al. [32] elaborated that the semantics of term was divided into two parts: one was inherited semantics, which was same as the semantics of its ancestors, and the other was extended semantics, which was special in itself. The extended IC of a term *t*_*i*_ from the term *t*_*j*_ is defined as:

7 $$ I{C}_{\mathrm{extended}}\left({t}_j\to {t}_i\right)=IC\left({t}_i\right)-IC\left({t}_j\right) $$

where *t*_*j*_ is the ancestor of *t*_*i*_. Furthermore, the extended IC of the term *t*_*i*_ from a term set *AS*(*t*_*i*_), *IC*_*extended*_(*AS*(*t*_*i*_) → *t*_*i*_) is formulated as

8 $$ I{C}_{extended}\left(AS\left({t}_i\right)\to {t}_i\right)=IC\left({t}_i\right)-IC\left(AS\left({t}_i\right)\right) $$

*AS*(*t*_*i*_) is the ancestor set of term *t*_*i*_. The Eq. (8) suggests that the term inherits all the semantics of its ancestors. In other words, a term transmits all its semantics to each descendant equally. Besides, Teng’s method doesn’t take into account the specificity of edges in the ontology. Obviously, this model doesn’t meet human perspective.

Vector-based methods represent each genes as a binary vector. Each GO term has the value 1 if it annotates gene or 0 otherwise [50]. Afterwards, Chaba et al. [51] made a further improvement that weighted the terms according to their IC values in the vector. A gene is represented by the following specific vector: *g* = (*w*_1_, *w*_2_ ⋯ *w*_*n*_) and *w*_*i*_ is the corresponding term IC. The functional similarity between two genes is given below:

9 $$ sim\left({g}_1,{g}_2\right)=\frac{\overrightarrow{g_1}\ast \overrightarrow{g_2}}{\sqrt{{\left|{g}_1\right|}^2+{\left|{g}_2\right|}^2}} $$

where ${\overrightarrow{g}}_1$ and ${\overrightarrow{g}}_2$ represent the corresponding vectors of gene *g*_1_ and *g*_2_. To our knowledge, vector-based methods ignore some valuable information which is implicit in the semantics and term relationships in the GO graph.

**Fig. 1:**
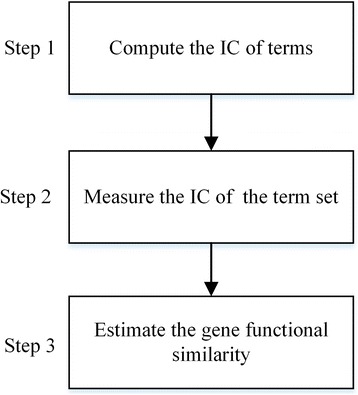
The framework of WIS

In summary, methods of gene functional similarity exist bias when they measure the IC of terms and term sets. Therefore, we propose a novel method called **Weighted Inherited Semantics** (WIS) for accuracy comparison of gene function. WIS computes the IC of a term by employing its depth, the number of its ancestors as well as the topology of its descendants in the GO graph. Secondly, WIS measures the IC of a term set by means of considering the weighted inherited semantics of terms. In the end, WIS estimates the gene functional similarity based on the IC overlap ratio of term sets. The computing framework of WIS is represented in Fig. 1.

## Results

### The distribution of term IC based on different models

**Fig. 2:**
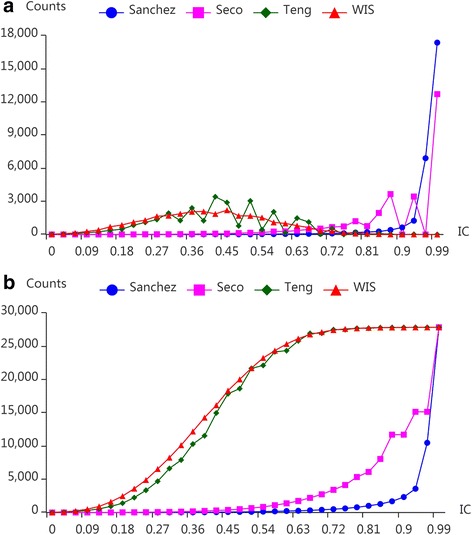
The distribution of term IC based on different models on BP ontology. **a** is the IC distribution on discrete points and **b** depicts the cumulative curves for IC distribution for each model

For the purpose of comparing the models intuitively which are Sanchez, Seco, Teng and WIS, the distribution of term IC based on BP ontology is given in Fig. 2. The results on CC and MF ontologies are presented by Additional file 1: Figure S1.

For Sanchez’s model, 87% of term IC is higher than 0.9. Only a small amount of term IC is varied between (0,0.9). The result of Seco has the similar problem. There is only 15% of term IC in range 0 to 0.7 totally. Therefore, these two models don’t show the specificity of different terms in the ontology. Hence, the distribution of term IC is very unreasonable. The results of Teng’s model have a great improvement comparing with the two models above. IC of terms is distributed in each interval reasonably. However, further analysis suggests IC of terms gathers at some points such as 0.39 and 0.42. By contrast, WIS has the ability to distribute the term IC in each interval evenly. The cumulative curve of WIS is smoother than Teng’s. This is because WIS makes the best use of the term information in the ontology and fully defines the specificity of a term. As a result, WIS performs better than other models in terms of the distribution of term IC (See the ‘Discussion’ section for details).

### Functional classification of genes in a biological pathway

**Fig. 3:**
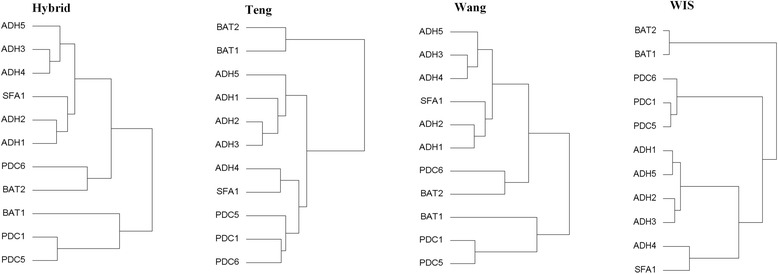
Clustering results of genes based on functional similarity values on MF ontology

We take the pathway ‘valine degradation’ as an example to examine the performance of WIS. As is shown in Table 1, there are total 11 genes which involve in 3 reactions in the selected pathway. The gene names and corresponding EC numbers in the pathway are presented. The functional similarity values among these genes are computed by WIS, Wang, Teng and Hybrid. The results of functional similarity are listed in Additional file 1: Table S1-S4 . The dendrograms generated by complete linkage hierarchical clustering of these genes using WIS and relevant measures are displayed in Fig. 3, respectively.

**Table 1:** Functions of genes in valine degradation pathway

Class ID	EC number	Gene Names
1	1.1.1.1	ADH1
	1.1.1.1	ADH2
	1.1.1.1	ADH3
	1.1.1.1	ADH4
	1.1.1.1	ADH5
	1.1.1.1	SFA1
2	2.6.1.42	BAT1
	2.6.1.42	BAT2
3	4.1.1.1	PDC1
	4.1.1.1	PDC5
	4.1.1.1	PDC6

As is demonstrated in Fig. 3, WIS has clustered the 11 genes into 3 clusters correctly. The first class contains gene SFA1, ADH1, ADH2, ADH3, ADH4 and ADH5, all of which have the similar subtype that EC number is 1.1.1.1. Meanwhile, PDC1, PDC5 and PDC6 are clustered into another group with the same EC number (4.1.1.1). BAT1 and BAT2 are clustered into the third group precisely. The result suggests that clustering result of WIS is consistent with the human perspective in functional classification of genes in the pathway.

In contrast, the clustering results obtained by relevant measures are mixed. For method Hybrid, it fails in the first level when it assigns high similarity to BTA2 and PDC6. For method Teng, since ADH4 has a higher similarity with SFA1 than PDC1, PDC5 and PDC6, these genes are not in their proper positions. As for method Wang, BAT1, PDC1 and PDC5 are grouped together in the first level. The clustering results are incorrect apparently. Therefore, functional similarities obtained by method Hybrid, Teng and Wang can’t characterize the gene functional relationship consistently with the human perspectives in the pathway.

### The results of CESSM

In order to evaluate effectiveness of the proposed method, the functional similarities of 13,430 protein pairs were computed by WIS as well as other tested methods. Considering GO aspects and electronic annotations may influence the performance of these methods, validation experiments are conducted on six GOAs: MF_IEA+, MF_IEA-, CC_IEA+, CC_IEA-, BP_IEA+ and BP_IEA-. The CESSM enables the comparison of WIS against 11 pairwise and groupwise functional similarity methods. We only compare WIS against six typical methods including simUI, simGIC, Teng as well as Resnik’s, Lin’s and Jiang and Conrath’s methods based on BMA rule, respectively. The experimental results on CC, BP and MF ontologies with IEA (IEA+) and without IEA (IEA-) are presented in Table 2. The best results are in bold.

**Table 2:** The performances of different methods on seven experiments

GOA	Metric	simGIC	simUI	Resnik	Lin	JC	Teng	WIS
MF_IEA+	ECC	0.6219	0.6365	0.6027	0.6417	0.5612	**0.6726**	0.6439
	Pfam	**0.638**	0.6181	0.5718	0.5639	0.4908	0.5765	0.6205
	Seq	**0.7172**	0.5925	0.6686	0.6063	0.5459	0.5943	0.6802
	Res	0.9559	0.9671	0.9577	0.5705	0.2409	0.9762	**0.9866**
BP_IEA+	ECC	0.3981	0.4022	0.4444	0.4352	0.3707	**0.4648**	0.4127
	Pfam	0.4574	0.4505	0.4587	0.3727	0.3318	0.4679	**0.4831**
	Seq	0.7732	0.7304	0.7397	0.6369	0.5864	0.7293	**0.7744**
	Res	0.8373	0.8628	0.9004	**0.9326**	0.3345	0.9076	0.8533
CC_IEA+	ECC	0.3613	0.3757	**0.3776**	0.3683	0.2598	0.3741	0.3702
	Pfam	0.4974	**0.5214**	0.493	0.485	0.3123	0.496	0.5056
	Seq	0.75	0.6721	0.7113	0.6398	0.5013	0.6549	**0.7561**
	Res	0.9001	0.9337	0.9167	0.9359	0.3098	**0.9371**	0.8926
MF_ IEA-	ECC	0.5874	0.5782	0.4841	0.5161	0.5189	**0.6502**	0.5947
	Pfam	0.5824	0.5504	0.5221	0.5148	0.4503	0.5703	**0.5865**
	Seq	0.6665	0.5907	0.6512	0.5976	0.5219	0.6443	**0.7032**
	Res	0.9358	0.9304	0.9335	0.9376	0.3641	**0.9605**	0.9345
BP_IEA-	ECC	0.3887	0.3818	0.4257	0.4216	0.4113	**0.4311**	0.3945
	Pfam	0.4383	0.4253	**0.4506**	0.381	0.274	0.4171	0.4325
	Seq	0.7359	0.6949	0.7267	0.6269	0.5333	0.6754	**0.7373**
	Res	0.8697	0.8831	0.8929	**0.9117**	0.3573	0.8966	0.8561
CC_IEA-	ECC	0.3502	**0.3527**	0.3443	0.339	0.2519	0.3512	0.3492
	Pfam	0.4681	**0.4872**	0.4676	0.4562	0.3321	0.4725	0.4781
	Seq	0.7348	0.6499	0.7214	0.6441	0.5013	0.6875	**0.7389**
	Res	0.8691	0.9072	0.8921	0.9102	0.3441	**0.911**	0.8686

The best results are in bold

As is shown in Table 2, there are totally 24 group experiments. As for SeqSim, WIS achieves the highest correlation in five out of six experiments except for MF_IEA+. Regarding Pfam, WIS wins first on BP_IEA+ and MF_IEA- experiments. Moreover, WIS gets the rank one on MF_IEA+ experiment of Res. In contrast, as for ECC, Teng shows the best correlation on MF_IEA+, BP_IEA+, MF_IEA- and BP_IEA- experiments. Teng also wins first on Res experiments of CC_IEA+, MF_IEA- and CC_IEA-. Additionally, simGIC and simUI achieve highest correlations in the corresponding experiments. For pairwise methods, Resnik and Lin only show highest correlations on four experiments in total.

At the same time, we also accumulate the correlations on ECC, SeqSim and Res for each method. Annotations with IEA and without IEA are both considered respectively. The performance of WIS is the best of the seven methods. For annotations with IEA, the sum of WIS is 5.2467 ranking first followed by simGIC and Resnik which are 5.2145 and 5.0678 respectively. For annotations without IEA, WIS also gets the rank one followed by simGIC and Teng. Detail results about the results are provided in Additional file 1: Table S5, Figure S2 and S3, available online.

In summary, WIS performs better than other six measures on MF, BP, CC ontology when they are evaluated on ECC, Pfam, SeqSim, resolutions, respectively. In all cases, WIS wins first on eight experiments followed by Teng and simUI methods. It is noteworthy that WIS and other groupwise methods (Teng, simUI, simGIC), in general, perform better than the pairwise methods (Resnik, Lin and JC) on CESSM.

### Protein-Protein interaction of yeast and human

Functional similarity between genes in yeast and human PPI datasets are computed by eleven measures which are Resnik, Jiang and Conrath, Lin, Wang, simGIC, simUI, Teng, ResnikGrasm [52], WangWV, simRel [7], and WIS. The pairwise approaches adopt the BMA rule to combine semantic similarity of terms. It is noteworthy that the original method in [12] is called method Wang. After taking our proposed weighting scheme, method Wang is called WangWV. The aim of adding WangWV is to compare the effectiveness of the proposed weighting scheme. Thereafter, we plot the ROC curves for each method and calculate the areas under the curves (AUC). At the same time, we also calculate F1-scores for different classification cut-off points for Resnik and WIS measures.

**Fig. 4:**
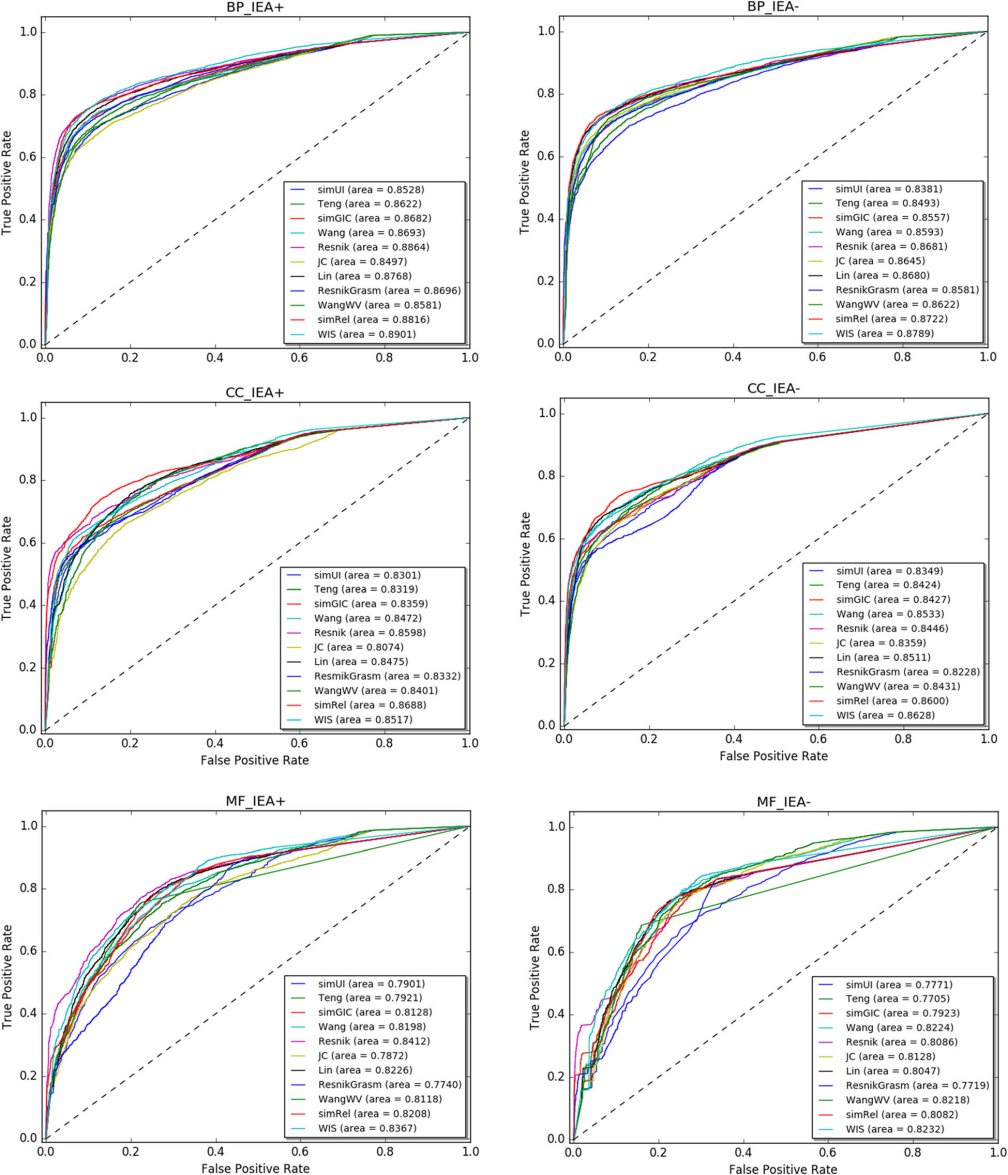
ROC curves for yeast PPI dataset. ROC evaluations of functional similarity measures at different cutoffs are shown. The evaluation was performed on CC, BP, MF ontologies. The BMA rule for pairwise approaches was used on the dataset, with electronic annotations (IEA+) and without electronic annotations (IEA-)

**Fig. 5:**
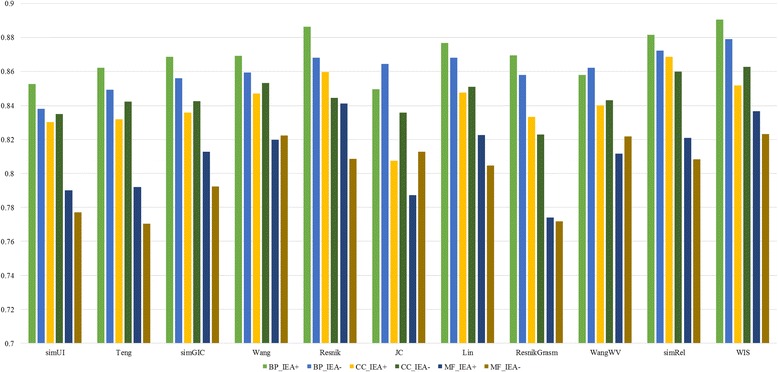
Barplot of AUCs of different measures on yeast PPI dataset for BP, CC and MF ontologies (IEA+ and IEA-)

ROC curves for each method in terms of BP, CC and MF ontologies on yeast PPI datasets are shown in Fig. 4. In order to facilitate the comparison of experimental results, the AUC for every method is listed in Table 3. The barplot of the AUC about different measures is represented in Fig. 5. WIS ranks first on four out of six experiments which are BP_IEA+, BP_ IEA-, CC_IEA- and MF_IEA-. Its advantage on correlations is quite prominent (>0.1). For example, the correlation of WIS is 0.8628 on CC_IEA- experiment, while the result for Resnik is 0.8446. Resnik ranks first on MF_IEA+ experiment and simRel gets the first on CC_IEA+ experiment. WIS and Resnik are neck and neck on CC_IEA+ experiment since the results of them are 0.8517 and 0.8598, respectively. Besides, the performance of Wang and WangWV is very close. This means that our proposed weighting scheme is reasonable since the fixed weights in [12] is selected according to a series of experiments. On the whole, pairwise approaches show relatively poor performance and only get the highest accuracy on two experiments. The groupwise approaches perform better than the pairwise approaches in this dataset.

**Table 3:** AUC of the functional similarity measures for three GOs using BMA in the PPI task on yeast dataset (IEA+ and IEA-)

Methods	BP_IEA+	BP_IEA-	CC_IEA+	CC_IEA-	MF_IEA+	MF_IEA-
simUI	0.8528	0.8381	0.8301	0.8349	0.7901	0.7771
Teng	0.8622	0.8493	0.8319	0.8424	0.7921	0.7705
simGIC	0.8688	0.8560	0.8359	0.8427	0.8128	0.7923
Wang	0.8693	0.8593	0.8472	0.8533	0.8198	0.8224
Resnik	0.8864	0.8681	0.8598	0.8446	**0.8412**	0.8086
JC	0.8497	0.8645	0.8074	0.8359	0.7872	0.8128
Lin	0.8768	0.8680	0.8475	0.8511	0.8226	0.8047
ResnikGrasm	0.8696	0.8581	0.8332	0.8228	0.7740	0.7719
WangWV	0.8581	0.8622	0.8401	0.8431	0.8118	0.8218
simRel	0.8816	0.8722	**0.8688**	0.8600	0.8208	0.8082
WIS	**0.8907**	**0.8792**	0.8517	**0.8628**	0.8367	**0.8232**

The best results are in bold

**Fig. 6:**
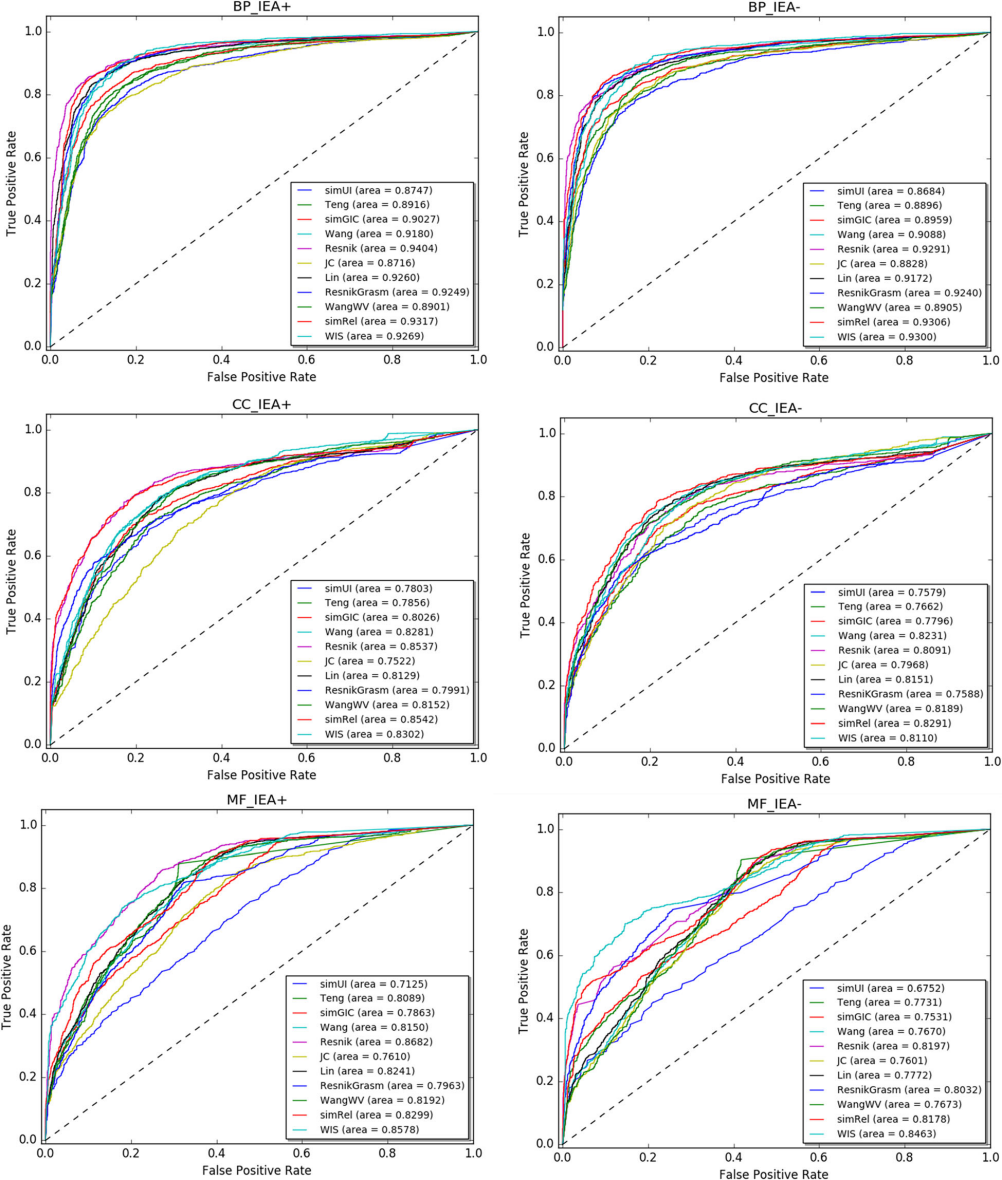
ROC curves for human PPI dataset. The evaluation was performed using CC, BP and MF ontologies. The BMA rule for pairwise approaches was used on the dataset, with electronic annotations (IEA+) and without electronic annotations (IEA-)

**Fig. 7:**
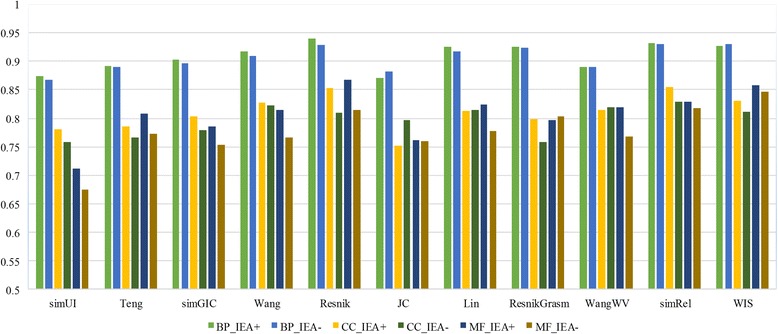
Barplot of AUCs of different measures on human PPI dataset in BP, CC and MF ontologies (IEA+ and IEA-)

The ROC curves of eleven methods on human datasets (with IEA+ and IEA-) are represented in Table 4, Figs. 6 and 7, respectively. As can be seen from the Table 4, simRel gets the best values on three out of six experiments which are BP_IEA-, CC_IEA+ and CC_IEA- experiments. Resnik ranks first on BP_IEA+ and MF_IEA+ experiments. WIS only ranks first on MF_IEA- experiment. Many authors have also found that Resnik performed the best performance [35, 47], for human PPI dataset (See discussion section). Although simRel performs best, further analysis shows that the performance between WIS and simRel is very close in some experiments. For instance, the AUC of simRel on BP_IEA- experiment is 0.9306, while the result of WIS is 0.9300. WIS wins second on two experiments which are BP_IEA- and MF_IEA+ experiments. The performance of Wang and WangWV is also very close in this dataset. What’s more, the performance of WIS is the best among the four groupwise approaches including simUI, simGIC and Teng. Corresponding with this, the results of simRel are the most prominent for pairwise approaches. WIS, simRel and Resnik show better ROC profiles for all three ontologies than other methods on the datasets.

**Table 4:** AUC of the functional similarity measures for three GOs using BMA in the PPI task on human dataset (IEA+ and IEA-)

Methods	BP_IEA+	BP_IEA-	CC_IEA+	CC_IEA-	MF_IEA+	MF_IEA-
simUI	0.8747	0.8684	0.7803	0.7579	0.7125	0.6752
Teng	0.8916	0.8896	0.7856	0.7662	0.8089	0.7731
simGIC	0.9027	0.8959	0.8026	0.7796	0.7863	0.7531
Wang	0.9180	0.9088	0.8281	0.8231	0.8150	0.7670
Resnik	**0.9404**	0.9291	0.8537	0.8091	**0.8682**	0.8150
JC	0.8716	0.8828	0.7522	0.7968	0.7610	0.7601
Lin	0.9260	0.9172	0.8129	0.8151	0.8241	0.7772
ResnikGrasm	0.9249	0.9240	0.7991	0.7588	0.7963	0.8032
WangWV	0.8901	0.8905	0.8152	0.8189	0.8192	0.7673
simRel	0.9317	**0.9306**	**0.8542**	**0.8291**	0.8299	0.8178
WIS	0.9269	0.9300	0.8302	0.8110	0.8578	**0.8463**

The best results are in bold

**Fig. 8:**
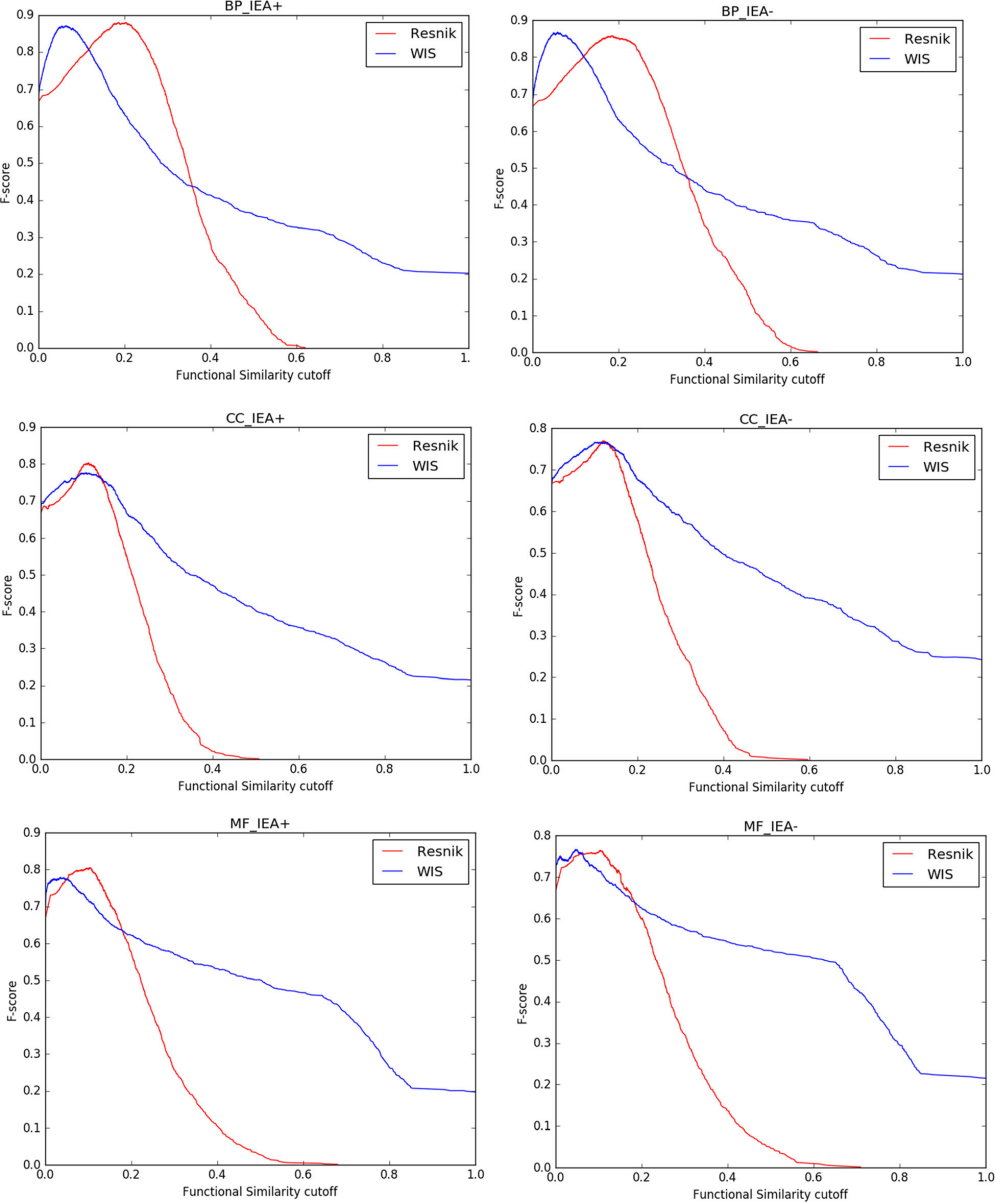
F1-score curves for yeast PPI dataset. F1-score (harmonic mean of precision and recall) evaluations of functional similarity measures at different cutoffs based on yeast PPI dataset are shown. Resnik and WIS were compared on BP, CC and MF ontologies with IEA+ and IEA- respectively. Resnik adopted BMA approach for combining multiple annotations

**Fig. 9:**
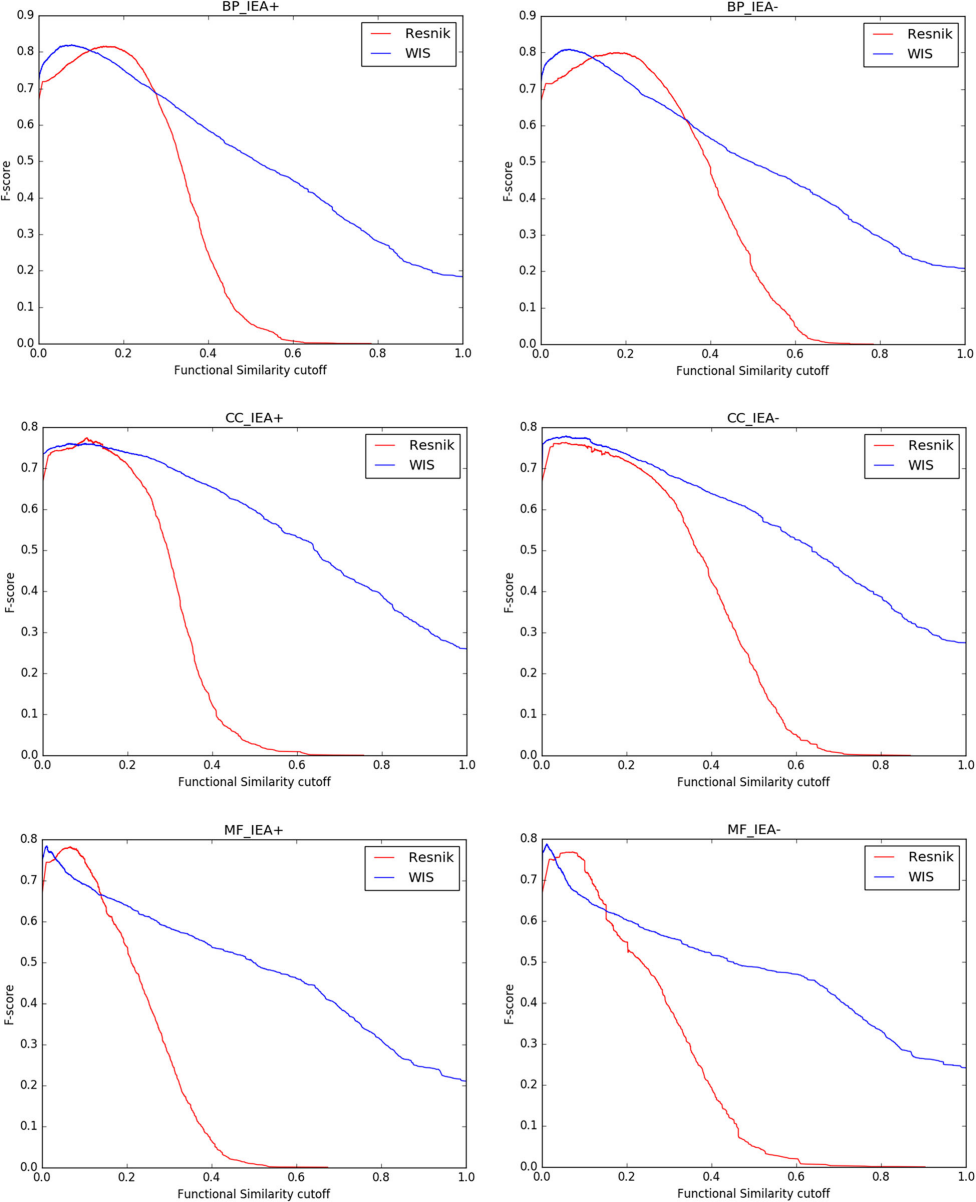
F1-score curves for human PPI dataset. F1 score (harmonic mean of precision and recall) evaluations of functional similarity measures at different cutoffs based on human PPI dataset are shown. Resnik and WIS were compared on BP, CC and MF ontologies with IEA+ and IEA- respectively. Resnik adopted BMA approach for combining multiple annotations

Because ROC curves are not always the only best approach to evaluate a classifier’s performance in a PPI task, we calculate F1-scores for different classification cut-off points for Resnik and WIS measures [53]. While the mean and maximum F1-scores can be indicators of one classifier’s performance in the detection of positive interactions which is similar to AUC, maximum F1-score also helps in selection of the best classification cut-off point of a classifier having its ROC curve. The F1-score curves on the datasets of yeast and human are shown in Figs. 8 and 9 respectively. The evaluation was performed on BP, CC and MF ontologies. Thereafter, the mean and maximum F1-score values on the two datasets were also calculated and the results were shown in Tables 5 and 6.

**Table 5:** F1-score of the Resnik and WIS measure for yeast PPI task (IEA+ and IEA-)

Type	Semantic measures	Mean of F1-score			Max of F1-score		
		BP	CC	MF	BP	CC	MF
IEA+	Resnik	0.6884	0.6388	0.6121	**0.8802**	**0.8029**	**0.8055**
	WIS	**0.7125**	**0.6594**	**0.6653**	0.8719	0.7762	0.7780
IEA-	Resnik	0.6818	0.6090	0.5569	0.8574	**0.7693**	0.7643
	WIS	**0.7174**	**0.6541**	**0.6669**	**0.8676**	0.7662	**0.7663**

The best results are in bold

**Table 6:** F1-score of the Resnik and WIS measure for human PPI task (IEA+ and IEA-)

Type	Semantic measures	Mean of F1-score			Max of F1-score		
		BP	CC	MF	BP	CC	MF
IEA+	Resnik	0.6661	0.6293	0.5900	0.8154	**0.7741**	0.7819
	WIS	**0.7051**	**0.6954**	**0.6630**	**0.8189**	0.7604	**0.7840**
IEA-	Resnik	0.6551	0.5905	0.4730	0.7998	0.7630	0.7678
	WIS	**0.7038**	**0.7035**	**0.6604**	**0.8087**	**0.7789**	**0.7875**

The best results are in bold

The performance of mean and max of F1-score on yeast dataset is shown in Table 5. The WIS prediction of PPIs based on the mean of F1-score is always better than the results achieved by Resnik. The mean F1-score of WIS is considerably higher than that of Resnik on both IEA+ and IEA- yeast datasets, while WIS doesn’t show great advantages against Resnik on max F1-score. In terms of max F1-score, Resnik achieves excellent performance on IEA+ datasets, while WIS is superior to Resnik on IEA- datasets of CC ontology only. For example, the max F1-scores on yeast IEA+ experiments for Resnik are 0.8802, 0.8029 and 0.8055 respectively, while results of WIS are 0.8717, 0.7762 and 0.7780.

The performances of mean and max of F1-score on human dataset are shown in Table 6. Resnik only win first on max of F1-score on CC_IEA+ experiment. WIS is superior to Resnik on all the rest of experiments. In summary, WIS outperforms other leading functional similarity methods including Resnik on yeast and human PPI datasets.

### Comparison analysis based on correlation with gene expression data

**Fig. 10:**
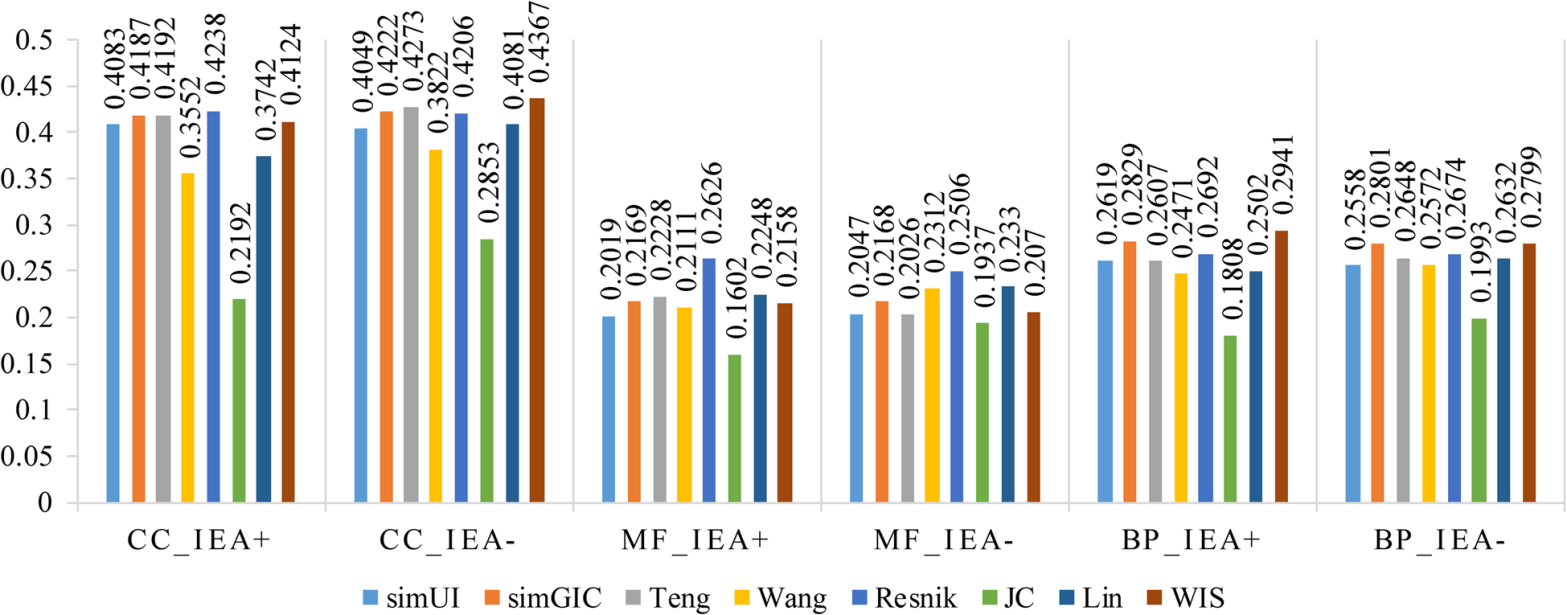
Barplot of Pearson’s correlation of functional similarity measures against gene expression data on three GOs (IEA+ and IEA-)

In this experiment, we will report Pearson’s correlation between gene expression data and functional similarity results which are from simGIC, simUI, Teng and pairwise measures based on BMA approach. Pearson’s correlation between gene expression and functional similarity for CC, BP and MF ontologies with IEAs (IEA+) and without IEAs (IEA-) is shown in Table 7 and Fig. 10.

**Table 7:** Pearson’s correlation of functional similarity measures for three GOs using BMA against gene expression data (IEA+ and IEA-)

Methods	CC_IEA+	CC_IEA-	MF_IEA+	MF_IEA-	BP_IEA+	BP_IEA-
simUI	0.4083	0.4049	0.2019	0.2047	0.2619	0.2558
simGIC	0.4187	0.4222	0.2169	0.2168	0.2829	**0.2801**
Teng	0.4192	0.4273	0.2228	0.2026	0.2607	0.2648
Wang	0.3552	0.3822	0.2111	0.2312	0.2471	0.2572
Resnik	**0.4238**	0.4206	**0.2626**	**0.2506**	0.2692	0.2674
JC	0.2192	0.2853	0.1602	0.1937	0.1808	0.1993
Lin	0.3742	0.4081	0.2248	0.2330	0.2502	0.2632
WIS	0.4124	**0.4367**	0.2158	0.2070	**0.2941**	0.2799

The best results are in bold

The CC ontology has the highest correlations in all cases, followed by BP and MF ontology. The experimental results show that Resnik generally outperforms other methods. As is demonstrated in Table 7, Resnik shows highest correlations on CC_IEA+, MF_IEA+ and MF_IEA- experiments, while WIS ranks first on BP_IEA+ and B P_IEA- experiments which are 0.4367 and 0.2941. Method Teng gets the highest correlations on CC_ IEA- experiment. Although WIS wins first on two experiments only and is inferior to Resnik which has been indicated to be better on yeast dataset by other authors [53, 54], its overall performance is better than other groupwise methods. Groupwise and pairwise methods show comparable performance in this dataset. WIS shows the best correlations (or one of the best) between gene expression and functional similarity with all three GO ontologies.

## Discussion

### The specificity of terms

How to measure the IC of terms reasonably is a controversial problem, but it is generally believed that the model should make the best use of term information and highlight its specificity. Therefore, a novel model for measuring the IC of a term is proposed. Comparing with other models, WIS considers not only the depths of terms, the number of their ancestors as well as the topology of their descendants in the GO graph. Therefore, our model has the ability to represents the specificity of terms to the maximum.

The characteristics of the four models have been listed in Table 8. These models do not rely on the corpus since they are all based on the structure of the GO graph. Sanchez considers the leaves and ancestors of terms, while Seco only considers the descendants of the terms. The term IC of Sanchez and Seco is mainly in the range of 0.9 to 1.0 and 0.75 to 1.0 respectively. As a result, the performance of Sanchez and Seco is extremely poor (See Fig. 2). Teng’s model uses the specificity and coverage of terms to measure their IC, but it ignores the ancestors of terms in the GO graph. WIS considers not only the depth of terms, but also the number of their ancestors and the topology of their descendants in the GO graph. As the results, our model performs best in representing the specificity of terms which is the foundation for measuring the IC of a term set accurately.

**Table 8:** Comparisons of IC computational models

Models	Whether the information of *t* affects the result				
	Corpus	Depth(*t*)	Descendant(*t*)	Leaves(*t*)	Ancestors(*t*)
Sanchez	No	No	No	Yes	Yes
Seco	No	No	Yes	No	No
Teng	No	Yes	Yes	No	No
WIS	No	Yes	Yes	No	Yes

Each model may employ different information of term *t* to measure its IC. We list out five types of information and discuss the characteristics of these four models above. “No” denotes that the factor has no effect on the model and vice versa. The corresponding explanation for each factor has been introduced in previous chapter

### The weighted inherited semantics between term and its parents

As is proposed by Teng, the semantics of a term is divided into two parts: one is inherited semantics, which is same as the semantics of its ancestors, and the other is extended semantics, which is special in itself. However, there is one serious drawback for Teng’s model. Because the edges in the GO graph are not always equal, the inherited semantics comes from its parents ought to have a weighted value according to the edge rather than is the same as the semantics of its ancestors.

In order to avoid repeated summing of term shared IC, WIS divides the semantics of a term into two part. One is weighted inherited semantics which is from its parents and the other is the extended semantics. Since WIS makes the best use of the relationship between terms, the results for measuring the annotating term set will be more reasonable. The results of WIS confirm that it is a effective and reliable way to estimate gene functional similarity.

### The difficulty on verifying the results

Since there is no direct way to ascertain the true functional similarity between two genes, how well a measure captures the similarity in function is not a trivial assessment [39]. For the sake of giving a comprehensive comparison, we select four group experiments to verify the performance of existing gene functional similarity methods.

The selected measures show different performances on different experiments. For example, groupwise methods outperform pairwise methods on CESSM dataset, while simRel performs best on human PPI experiments which is followed by WIS. The reason of this problem maybe the characteristics of different data sets. The proteins in CESSM are all well annotated. In contrast, the data set of yeast only considers the high quality interactions, but ignores the annotation richness for genes. Therefore, the number of annotations per gene is crucial to the performance of functional similarity measures. Besides, due to lack of the authority and uniform evaluation criteria, there are still existing some problems in comparing these methods objectively. Therefore, how to measure the functional similarity reliably is still a meaningful research area.

On PPI classification of yeast and human datasets, as we can see the results in Tables 3 and 4, Resnik also get high AUC values. As is known to us, current GOA database is incomplete and many proteins are only annotated with one or two GO terms. What’s more, these proteins which are not well studied are annotated with more general terms (near the root of the ontology). In this situation, if two proteins are annotated with the same GO terms, the functional similarity between the proteins calculated by most methods is always 1.0. Obviously, this is not meet human’s perspective. As a result, the methods that cannot distinguish the identical annotations may not perform well. As for the eleven methods listed in Table 4, three pairwise methods can distinguish the identical annotation, which are Resnik, simRel and ResnikGrasm. From the results, we can fortunately find that these three pairwise methods indeed perform better than the other methods which cannot distinguish the identical annotation. We can conduct other experiments and assess the performance of Resnik and sim Rel, and then further give a strong evidence.

In future work, WIS can be evaluated on human miRNA target gene sets and correlation with sequence similarity dataset. Then WIS also needs to be verified on other model organism that have high quality biological data. Since annotation richness is crucial to the performance of functional similarity methods, WIS should be investigated on datasets with different annotation richness. In the end, there may be some scope for improving the proposed measure on studying the specificity of terms and measuring the IC of a term set more reasonable.

## Conclusions

We proposed a novel method, namely WIS, to measure gene functional similarity based on GO. It is extensively evaluated on four different experiments which are functional classification of genes in biological pathway, CESSM dataset, protein–protein interaction prediction and correlation with gene expression. The experimental results suggest that WIS is a more effective and reliable way to estimate gene functional similarity comparing with the other tested methods. WIS has the following advantages.

First, WIS makes the best use of term information in the GO graph. WIS measures the IC of a term by considering its depth, the number of its ancestors and the topology of its descendants in the ontology. As a result, WIS can conquer the limitation of corpus bias, which affects the corpus-based approach heavily. Therefore, WIS can also fully measure the specificity of terms more objectively than other methods.

Second, WIS measures the IC of a term set by combining the inherited and extended IC of terms. Inherited IC is the weighted semantics which is from its parents and extended IC is special in itself. WIS considers the two types of semantics, so it can effectively avoid repeated summing of term shared IC, which is the key point for estimating the IC of a term set reasonably and correctly.

Third, WIS is very promising since it outperforms most existing state-of-the-art methods on all kinds of experiments. Pairwise approaches are sensitive to the number of annotations per gene since they are based on the combination of similarities between term pairs. In contrast, groupwise approaches are sensitive to the specificity of terms because they estimate gene functional similarity by comparing the terms in groups. Since WIS can measure the IC of terms and term sets more reasonably, the performance of WIS is more stability than other tested methods on the experiments. Therefore, it is an effective and reliable way to estimate gene functional similarity. The online service of WIS is available at http://nclab.hit.edu.cn/WIS freely.

## Methods

### Measure the IC of a term

Inspired by Sanchez and Teng’s model, a novel model for measuring the IC of a term is proposed. It is generally accepted that the deeper a term is, the more information it conveys. Terms with more ancestors will be more specific than terms with less ones. Besides, since IC gives a measure how specific a term is, we assume that the specificity of terms not only depend on their depths, but also have a relationship with the number of their ancestors as well as the topology of their descendants. Therefore, in order to fully define the specificity of a term, a novel computing model is given by

10 $$ IC(t)= depth(t)* \log \left(\left|AS(t)\right|\right)*\left(1-\frac{ \log \left({\displaystyle {\sum}_{a\in DS(t)}\frac{1}{depth(a)}+1}\right)}{ \log \left( \max \_ nodes\right)}\right) $$

where depth(*t*) denotes the depth of term *t* in the GO graph, AS(*t*) represents the ancestor set of term *t*, DS(*t*) denotes the descendants set of term *t* including *t* itself and max_nodes denotes the total number of terms in the GO ontology. The proposed model meets the requirement that IC of terms monotonically increases as terms move down in the ontology.

### Measure the IC of a term set by means of considering weighted inherited semantics of terms

First of all, we define the weight ω between a term *t* and its parent *t*_*p*_. The ω should be greater than 0 and less than 1 and can be formulated as

11 $$ \omega =\frac{Dst(t)}{Dst\left({t}_p\right)} $$

where *Dst*(*t*) is the number of descendants of term *t*. It should be noted that the number of term descendants is calculated using the DAG of the entire GO rather than the sub-graphs of term *t* [2]. The weight ω is invariable, except in cases of the deletion of obsolete terms or the addition of new terms accompanying the update of GO database.

Then, for the sake of measuring the IC of a term set, we take full account of the term IC as well as the weighted inherited semantics between terms. As a result, the semantics of a term is divided into two parts: one is weighted inherited semantics from its parents, and the other is extended semantics which is special in itself.

Suppose there is a term set *T* that only contains two terms, namely *t*_1_ and *t*_2_. Term *t*_1_ is the parent of *t*_2_. The weighted inherited semantics of *t*_2_ which comes from *t*_1_ is

12 $$ I{C}_{inherited}\left({t}_2\to {t}_1\right)={\omega}_{12}\ast IC\left({t}_1\right) $$

where the ω_12_ is the weighted value between term *t*_1_ and *t*_2_ and can be calculated using Equation (11). The extended IC of term *t*_2_ as for term *t*_1_ is defined as

13 $$ I{C}_{extended}\left({t}_2\to {t}_1\right)=IC\left({t}_2\right)-I{C}_{inherited}\left({t}_2\to {t}_1\right)=IC\left({t}_2\right)-{\omega}_{12}\ast IC\left({t}_1\right) $$

As a result, the IC of a term set *T* contains *t*_1_ and *t*_2_ is achieved by

$$ IC(T)=IC\left({t}_1\right)+IC\left({t}_2\right)=IC\left({t}_1\right)+I{C}_{\mathrm{extended}}\left({t}_2\to {t}_1\right)=IC\left({t}_1\right)+IC\left({t}_2\right)-{\omega}_{12}*IC\left({t}_1\right) $$

In this way, WIS can effectively avoid repeated summing of term shared IC.

### Example: measure the IC of a term set based on WIS

**Fig. 11:**
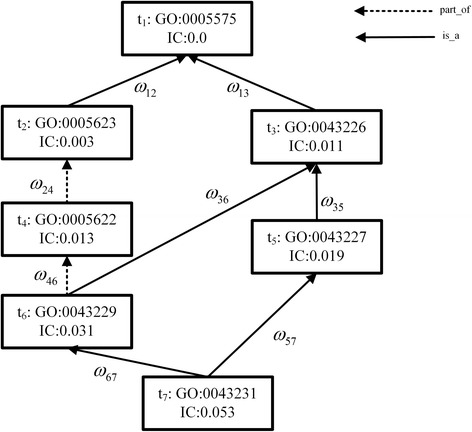
DAG for GO term *Intracellular Membrane-bound Organelle*: 0043231.

Suppose there is a term set *S* contains all terms in the Fig. 11. The IC of each term is also presented in Fig. 11 and the weight values of corresponding edges are shown in Table 9. We will take the set *S* as an example to demonstrate how to measure the IC of a term set based on WIS. The computational process for measuring the IC of term set S based on WIS is shown in Table 10.

**Table 9:** The weight values of corresponding edges in Figure 11

Edge	ω12	ω13	ω24	ω46	ω36	ω35	ω67	ω57
Value	0.80	0.46	0.57	0.71	0.92	0.70	0.73	0.96

**Table 10:** The computational process for measuring the IC of term set *S*

Step	Elements in S	IC(S)	Add element	ICextended	IC(S) + ICextended
1		0	*t*1	0	0
2	*t*1	0	*t*2	*IC*(*t*_2_) − *ω*_12_ ∗ *IC*(*t*_1_) = 0.003	0.003
3	*t*1,*t*2	0.003	*t*3	*IC*(*t*_3_) − *ω*_13_ ∗ *IC*(*t*_1_) = 0.011	0.014
4	*t*1,*t*2,*t*3	0.014	*t*4	*IC*(*t*_4_) − *ω*_24_ ∗ *IC*(*t*_2_) = 0.010	0.024
5	*t*1,*t*2,*t*3,*t*4	0.024	*t*5	*IC*(*t*_5_) − *ω*_35_ ∗ *IC*(*t*_3_) = 0.012	0.036
6	*t*1,*t*2,*t*3,*t*4,*t*5	0.036	*t*6	*IC*(*t*_6_) − *ω*_46_ ∗ *IC*(*t*_4_) − *ω*_36_ ∗ *IC*(*t*_3_) = 0.012	0.048
7	*t*1,*t*2,*t*3,*t*4,*t*5,*t*6	0.048	*t*7	*IC*(*t*_7_) − *ω*_67_ ∗ *IC*(*t*_6_) − *ω*_57_ ∗ *IC*(*t*_5_) = 0.003	0.051
8	*t*1,*t*2,*t*3,*t*4,*t*5,*t*6,*t*7	0.051		0	0.051

In step 1, term set *S* is null, and IC(*S*) is 0. Then we add the first term *t*_1_ into S. According to Equation (13), IC_extend_ is 0. Therefore, the last result for step one equals to IC(S) + IC_extended_ and is 0.

In step 2, term set S contains *t*_1_ only, and IC(S) is 0. We add the second term *t*_2_ into S. According to Equation (13), *IC*_*extend*_(*t*_2_ → *t*_1_) is 0.003. Therefore, the last result for step 2 equals to IC(S) + IC_extended_ and is 0.003.

**Fig. 12:**
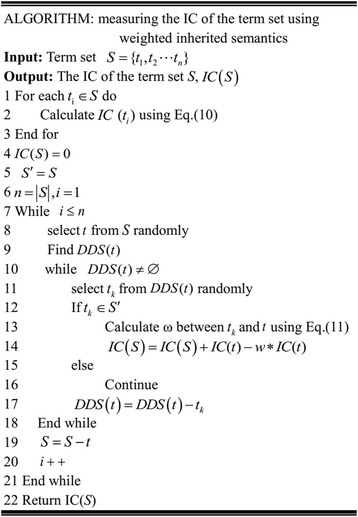
Algorithm for measuring the IC of a term set

The computational process of step 3 to 7 is similar to the step 2 and we don’t repeat here anymore. In step 8, all the terms have been added into term *S* already, the IC of set *S* is 0.051. The calculation for measuring the IC of *S* is finished. The detail algorithm for measuring the IC of a term set using WIS is described in Fig. 12.

### Measure the gene functional similarity between two genes based on WIS

Suppose gene *g*_1_ and *g*_2_ are annotated with term sets *T*_*g*1_ = {*t*_1_, *t*_2_ ⋯ *t*_*m*_} and *T*_*g*2_ = {*t*_1_, *t*_2_ ⋯ *t*_*n*_} respectively. Then, the functional similarity between *g*_1_ and *g*_2_ is given by

$$ Fu{n}_{sim}\left({g}_1,{g}_2\right)=\frac{IC\left({T}_{g1}\cap {T}_{g2}\right)}{IC\left({T}_{g1}\cup {T}_{g2}\right)} $$

where *T*_*g*1_ ∩ *T*_*g*2_ is the intersection of *T*_*g*1_ and *T*_*g*2_ and *T*_*g*1_ ∪ *T*_*g*2_ is the union of *T*_*g*1_ and *T*_*g*2_. The IC of corresponding term sets can be obtained by WIS.

### Experimental data and evaluation of the proposed approach

How well a measure captures the function similarity between two genes is not a trivial assessment because there is no direct way to ascertain the true functional similarity between them [2, 39]. However, the performance of existing functional similarity measurements can be verified in terms of pathway gene clustering [12, 55], correlations with sequence similarity [13, 46], gene expression profiling [41], protein-protein interactions [14, 54] and so on. In this article, the performance of WIS will be validated on four group experiments which are biological pathways of yeast, CESSM dataset, protein-protein interaction dataset of yeast and human as well as gene expression data of yeast. Additionally, it is noteworthy that pairwise approaches adopt the BMA rule to combine semantic similarity of terms since it is the best for evaluation of functional similarity measures.

### Gene Ontology data

We downloaded the Gene Ontology data from the Gene Ontology database (dated August 2015) containing 41,624 ontology terms totally subdivided into 3717 cellular component, 27,864 biological process and 9943 molecular function terms. Gene annotations for GO terms were downloaded from the Gene Ontology database for *S. cerevisiae* and H. Sapiens (dated October 2015).

### Biological pathway of yeast

Genes participate in a certain biological pathway may involve in several different molecular functions. They are endowed with different Enzyme Commission (EC) numbers according to the subtype of reaction that they catalyze at the molecular level. Therefore, it is an effective way to classify the genes according to their molecular functions of genes and validate the accuracy of functional similarity methods. If the clustering results are consistent with the artificial classification results based on the biological reactions, the measure is effective in charactering the functional similarity between genes [55]. Therefore, we have taken a few pathways from yeast pathway database (http://pathway.yeastgenome.org/) and the validated results are demonstrated for the valine degradation pathway only due to the space limitation.

### CESSM Dataset

We use the CESSM [56] tool to compare WIS with other leading methods. CESSM is a widely used platform which provides a standard dataset. It consists of 13,430 pairs of proteins involving 1039 distinct proteins and implements 11 state-of-the-art semantic similarity measures. We only consider the best-match average (BMA) rule of Resnik's, Lin's and Jiang and Conrath’s methods, coupled with simGIC, simUI and Teng [32]. It provides Pearson correlations with sequence similarity (Seq), protein family similarity (Pfam), enzyme commission classification similarity (ECC) and Resolution (Res) to evaluate these measures [32]. SeqSim is computed using a relative measure of sequence similarity based on the BLAST bitscores, which is called RRBS method [13]. The similarity between two proteins is computed by dividing the sum of the reciprocal BLAST bit scores by the sum of their dependent BLAST bitscores. The value of SeqSim ranges from 0 to 1.0. ECC is calculated using EC class similarity of proteins. According to [57], the value of ECC is between 0 and 4 that corresponds to the number of EC digits two proteins share. Pfam is measured via Jaccard similarity, where the similarity between proteins is the ratio between the number of domains they share and the total number of those they have. Resolution is the relative intensity with which values in the sequence similarity scale are translated into the semantic similarity. Resolution depicts the ability of a method to distinguish different levels of sequence similarity. Higher correlation and resolution values support the efficiency of the measures. A detail explanation for these criterions has been discussed by Pesquita [56].

### Protein-Protein interaction data of Yeast and Human

We collect protein-protein interaction (PPI) datasets of yeast and human from the Jain and Davis’s database [53, 58]. The database has around 3800 yeast PPIs and 1500 human PPIs which are core set of DIP yeast database (dated 2009) [14]. Negative datasets with the same number of PPIs for yeast and human are independently generated by randomly choosing annotated gene pairs for BP, CC and MF ontology, which are absent from a combined dataset of all possible PPIs [58, 59]. We conducted out experiments using the same data in [53]. In order to draw the ROC plots, the threshold of the functional similarity values between all gene pairs is varied between (0,1). The gene pairs with similarity values greater than the threshold are predicted to be positives, while those below the threshold are predicted to be negatives. Thereafter, the true positive and true negative, and false positive and false negative values are computed, and ROC curves can be plotted [14]. The area under the curve (AUC) obtained from the ROC plots is used to compare the performance of WIS against the other functional similarity measures. The F1-scores are also calculated for the corresponding measures.

### Gene expression data for yeast

Correlation between gene expression and gene functional similarity is another desirable criterion since many gene products that participate in the same biological process or are functionally related have similar expression profiles [41]. Therefore, the comparison of expression similarity and functional similarity between genes can be used as a standard performance evaluation. Methods having higher correlation will be regard as a better performance. The gene expression dataset for S.cerevisiae comes from Jain and Davis [53]. The dataset contains 5000 *S. cerevisiae* gene pairs randomly selected from a list of all possible pairs of proteins in the gene expression dataset [58]. We use all 5000 gene pairs from their study and consider genes with electronic annotations (IEA+) and non-electronic annotations (IEA-).

## Additional file

Additional file 1: In the additional file, **Figure S1.** depecits the distributions of term IC based on CC and MF ontology for each IC calculation model. **Tables S1-S4.** show the functional similarity values between seleted genes for each method on the functional classification of genes in a biological pathway experiment. **Table S5**, **Figure S2.** and **Figure S3.** list out the accumulated results about correlations on ECC, SeqSim and Res for each method with IEA+ and IEA- on CESSM datasets. The more detail descriptions about the experimental results are shown in the Additional file 1. (PDF 448 kb)

## References

[1] Ashburner M, Ball CA, Blake JA, Botstein D, Butler H, Cherry JM, Davis AP, Dolinski K, Dwight SS, Eppig JT (2000). Gene Ontology: tool for the unification of biology. Nature genetics.

[2] Xu Y, Guo M, Shi W, Liu X, Wang C (2013). A novel insight into Gene Ontology semantic similarity. Genomics.

[3] Bairoch AM, Apweiler R, Wu CH, Barker WC, Boeckmann B, Ferro Rojas S, Gasteiger E, Huang H, Lopez R, Magrane M (2005). The universal protein resource (UniProt). Nucleic acids research.

[4] Kriventseva EV, Fleischmann W, Zdobnov EM, Apweiler R (2001). CluSTr: a database of clusters of SWISS-PROT+ TrEMBL proteins. Nucleic acids research.

[5] Song X, Li L, Srimani PK, Yu PS, Wang JZ (2014). Measure the semantic similarity of go terms using aggregate information content. IEEE/ACM Transactions on Computational Biology and Bioinformatics (TCBB).

[6] Peng J, Wang T, Wang J, Wang Y, Chen J (2016). Extending gene ontology with gene association networks. Bioinformatics.

[7] Schlicker A, Domingues FS, Rahnenführer J, Lengauer T (2006). A new measure for functional similarity of gene products based on Gene Ontology. BMC bioinformatics.

[8] Schlicker A, Lengauer T, Albrecht M (2010). Improving disease gene prioritization using the semantic similarity of Gene Ontology terms. Bioinformatics.

[9] Jiang JJ, Conrath DW (1997). Semantic similarity based on corpus statistics and lexical taxonomy.

[10] Lin D (1998). An information-theoretic definition of similarity. ICML.

[11] Resnik P (1999). Semantic similarity in a taxonomy: An information-based measure and its application to problems of ambiguity in natural language. J Artif Intell Res.

[12] Wang JZ, Du Z, Payattakool R, Philip SY, Chen C-F (2007). A new method to measure the semantic similarity of GO terms. Bioinformatics.

[13] Pesquita C, Faria D, Bastos H, Ferreira AE, Falcão AO, Couto FM (2008). Metrics for GO based protein semantic similarity: a systematic evaluation. BMC bioinformatics.

[14] Bandyopadhyay S, Mallick K (2014). A New Path Based Hybrid Measure for Gene Ontology Similarity. Ieee-Acm Transactions on Computational Biology and Bioinformatics.

[15] Wu H, Su Z, Mao F, Olman V, Xu Y (2005). Prediction of functional modules based on comparative genome analysis and Gene Ontology application. Nucleic acids research.

[16] Cheng J, Cline M, Martin J, Finkelstein D, Awad T, Kulp D, Siani-Rose MA (2004). A knowledge-based clustering algorithm driven by gene ontology. Journal of biopharmaceutical statistics.

[17] Li M, Wu X, Pan Y, Wang J (2013). hF‐measure: A new measurement for evaluating clusters in protein–protein interaction networks. Proteomics.

[18] Smyth GK. Limma: linear models for microarray data. Bioinformatics and computational biology solutions using R and Bioconductor Springer. 2005;397–420.

[19] Pekar V, Staab S. Taxonomy learning: factoring the structure of a taxonomy into a semantic classification decision. In: Proceedings of the 19th international conference on Computational linguistics-Volume 1: 2002. Association for Computational Linguistics: 1–7.

[20] Brameier M, Wiuf C (2007). Co-clustering and visualization of gene expression data and gene ontology terms for Saccharomyces cerevisiae using self-organizing maps. Journal of biomedical informatics.

[21] Cho YR, Zhang AD, Xu X (2009). Semantic similarity based feature extraction from microarray expression data. Int J Data Min Bioin.

[22] Yang D, Li YH, Xiao H, Liu Q, Zhang M, Zhu J, Ma WC, Yao C, Wang J, Wang D (2008). Gaining confidence in biological interpretation of the microarray data: the functional consistence of the significant GO categories. Bioinformatics.

[23] Qu Y, Xu S. Supervised cluster analysis for microarray data based on multivariate Gaussian mixture[J]. Bioinformatics, 2004, 20(12):1905–1913. 10.1093/bioinformatics/bth177 15044244

[24] Lee PH, Lee D (2005). Modularized learning of genetic interaction networks from biological annotations and mRNA expression data. Bioinformatics.

[25] Yu G, Fu G, Wang J, Zhu H. Predicting Protein Function via Semantic Integration of Multiple Networks. IEEE/ACM Transactions on Computational Biology and Bioinformatics. 13;(2):220–232. 10.1109/TCBB.2015.2459713 26800544

[26] Yu G, Zhu H, Domeniconi C. Predicting protein functions using incomplete hierarchical labels. BMC Bioinformatics. 2015;16(1). 10.1186/s12859-014-0430-y PMC4384381 25591917

[27] Lei Z, Dai Y (2006). Assessing protein similarity with Gene Ontology and its use in subnuclear localization prediction. BMC bioinformatics.

[28] Cheng L, Li J, Ju P, Peng J, Wang Y (2014). SemFunSim: a new method for measuring disease similarity by integrating semantic and gene functional association.

[29] Chen J, Aronow BJ, Jegga AG (2009). Disease candidate gene identification and prioritization using protein interaction networks. BMC bioinformatics.

[30] Guo X, Liu R, Shriver CD, Hu H, Liebman MN (2006). Assessing semantic similarity measures for the characterization of human regulatory pathways. Bioinformatics.

[31] Tuikkala J, Elo L, Nevalainen OS, Aittokallio T (2006). Improving missing value estimation in microarray data with gene ontology. Bioinformatics.

[32] Teng Z, Guo M, Liu X, Dai Q, Wang C, Xuan P (2013). Measuring gene functional similarity based on group-wise comparison of GO terms. Bioinformatics.

[33] Peng J, Wang T, Hu J, Wang Y, Chen J (2016). Constructing Networks of Organelle Functional Modules in Arabidopsis. Current Genomics..

[34] Seco N, Veale T, Hayes J. An intrinsic information content metric for semantic similarity in WordNet[C]. ECAI. 2004;16:1089.

[35] Harispe S, Sánchez D, Ranwez S, Janaqi S, Montmain J (2014). A framework for unifying ontology-based semantic similarity measures: A study in the biomedical domain. Journal of biomedical informatics.

[36] Sánchez D, Batet M (2011). Semantic similarity estimation in the biomedical domain: An ontology-based information-theoretic perspective. Journal of biomedical informatics.

[37] Sánchez D, Batet M, Isern D (2011). Ontology-based information content computation. Knowledge-Based Systems.

[38] Guzzi PH, Mina M, Guerra C, Cannataro M (2012). Semantic similarity analysis of protein data: assessment with biological features and issues. Briefings in bioinformatics.

[39] Pesquita C, Faria D, Falcao AO, Lord P, Couto FM (2009). Semantic similarity in biomedical ontologies. PLoS computational biology.

[40] Couto FM, Silva MJ, Coutinho PM: Semantic similarity over the gene ontology: family correlation and selecting disjunctive ancestors. In: Proceedings of the 14th ACM international conference on Information and knowledge management: 2005. ACM: 343-344.

[41] Sevilla JL, Segura V, Podhorski A, Guruceaga E, Mato JM, Martinez-Cruz LA, Corrales FJ, Rubio A (2005). Correlation between gene expression and GO semantic similarity. Computational Biology and Bioinformatics, IEEE/ACM Transactions on.

[42] Yu H, Gao L, Tu K, Guo Z (2005). Broadly predicting specific gene functions with expression similarity and taxonomy similarity. Gene.

[43] Del Pozo A, Pazos F, Valencia A (2008). Defining functional distances over gene ontology. BMC bioinformatics.

[44] Othman RM, Deris S, Illias RM (2008). A genetic similarity algorithm for searching the Gene Ontology terms and annotating anonymous protein sequences. Journal of biomedical informatics.

[45] Shen Y, Zhang S, Wong H-S: A new method for measuring the semantic similarity on Gene Ontology. In: Bioinformatics and Biomedicine (BIBM), 2010 IEEE International Conference on: 2010. IEEE. pp. 533-8.

[46] Mistry M, Pavlidis P (2008). Gene Ontology term overlap as a measure of gene functional similarity. BMC bioinformatics.

[47] Tversky A (1977). Features of similarity. Psychological review.

[48] Lee HK, Hsu AK, Sajdak J, Qin J, Pavlidis P (2004). Coexpression analysis of human genes across many microarray data sets. Genome research.

[49] Pesquita C, Faria D, Bastos H, Falcão A, Couto F. Evaluating GO-based semantic similarity measures. In: Proc 10th Annual Bio-Ontologies Meeting: 2007. 38.

[50] Alvord G, Roayaei J, Stephens R, Baseler MW, Lane HC, Lempicki RA (2007). The DAVID Gene Functional Classification Tool: a novel biological module-centric algorithm to functionally analyze large gene lists. Genome biology.

[51] Chabalier J, Mosser J, Burgun A (2007). A transversal approach to predict gene product networks from ontology-based similarity. BMC bioinformatics.

[52] Couto FM, Silva MJ, Coutinho PM (2007). Measuring semantic similarity between Gene Ontology terms. Data & knowledge engineering.

[53] Jain S, Bader GD (2010). An improved method for scoring protein-protein interactions using semantic similarity within the gene ontology. BMC bioinformatics.

[54] Xu T, Du L, Zhou Y (2008). Evaluation of GO-based functional similarity measures using S. cerevisiae protein interaction and expression profile data. BMC bioinformatics.

[55] Zhang S-B, Lai J-H. A hybrid measure for the semantic similarity of gene ontology terms. In: Systems and Informatics (ICSAI), 2014 2nd International Conference on: 2014. IEEE: 911-6.

[56] Pesquita C, Pessoa D, Faria D, Couto F (2009). CESSM: Collaborative evaluation of semantic similarity measures. JB2009: Challenges in Bioinformatics.

[57] Devos D, Valencia A (2000). Practical limits of function prediction. Proteins: Structure, Function, and Bioinformatics.

[58] Pesaranghader A, Matwin S, Sokolova M, Beiko RG (2016). simDEF: definition-based semantic similarity measure of gene ontology terms for functional similarity analysis of genes. Bioinformatics.

[59] Razick S, Magklaras G, Donaldson IM (2008). iRefIndex: a consolidated protein interaction database with provenance. BMC bioinformatics.

