# Supplementary material for: An improved method for functional similarity analysis of genes based on Gene Ontology
Source: BMC Syst Biol. 2016 Dec 23;10(Suppl 4):119. doi: 10.1186/s12918-016-0359-z (PMC5259995; doi:10.1186/s12918-016-0359-z)

Additional file 1

Supplementary Figure S1-The distribution of term IC on CC and MF ontology

The distribution of term IC in each interval is depicted in Figure S1(a) and Figure S1(b) for CC and MF respectively. The accumulated results of term IC on CC and MF ontology are listed in Figure S1(c) and Figure S1(d). From the result, we can find that term IC can be distributed better by WIS than other models. The results achieve a great improvement by WIS.

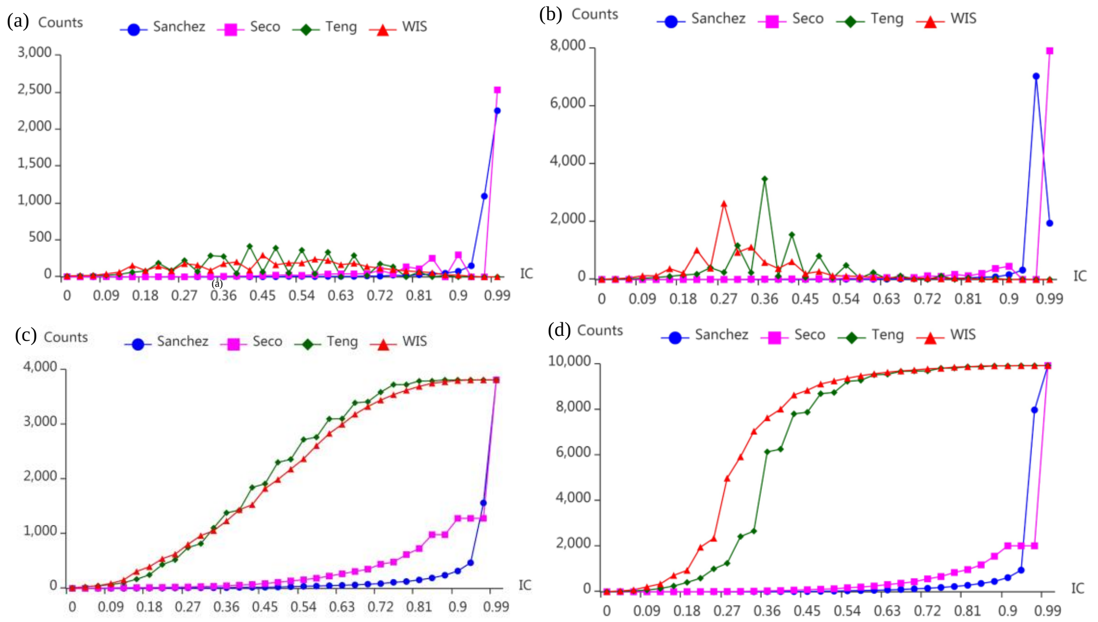

CC MF

Supplementary Table S1-4. Functional classification of genes in a biological pathway

Table S1-4 list out the functional similarity values among genes in *valine degradation* pathway for each method. And we obtain four dendrograms respectively using the data by complete linkage hierarchical clustering of these genes. Our method WIS performs best among the four typical approaches.

Supplementary Table S1. Similarity values among genes in *valine degradation* pathway obtained by method Hybrid on BP ontology

	ADH3	ADH4	ADH5	ADH2	ADH1	SFA1	PDC1	PDC5	PDC6	BAT1	BAT2
ADH3	1.00	0.90	0.85	0.90	0.84	0.92	0.58	0.53	0.65	0.62	0.65
ADH4	0.90	1.00	0.82	0.81	0.82	0.84	0.58	0.56	0.67	0.65	0.67
ADH5	0.85	0.82	1.00	0.85	0.79	0.87	0.55	0.56	0.68	0.69	0.67
ADH2	0.90	0.81	0.85	1.00	0.89	0.91	0.53	0.57	0.72	0.66	0.72
ADH1	0.84	0.82	0.79	0.89	1.00	0.88	0.51	0.55	0.70	0.65	0.68
SFA1	0.92	0.84	0.87	0.91	0.88	1.00	0.51	0.54	0.67	0.70	0.66
PDC1	0.58	0.58	0.55	0.53	0.51	0.51	1.00	0.96	0.63	0.60	0.63
PDC5	0.53	0.56	0.56	0.57	0.55	0.54	0.96	1.00	0.67	0.62	0.67
PDC6	0.65	0.67	0.68	0.72	0.70	0.67	0.63	0.67	1.00	0.91	0.94
BAT1	0.62	0.65	0.69	0.66	0.65	0.70	0.60	0.62	0.91	1.00	0.92
BAT2	0.65	0.67	0.67	0.72	0.68	0.66	0.63	0.67	0.94	0.92	1.00

Supplementary Table S2. Similarity values among genes in *valine degradation* pathway obtained by method Teng on BP ontology

	ADH2	ADH1	ADH4	SFA1	ADH3	ADH5	PDC1	PDC6	PDC5	BAT2	BAT1
ADH2	1.00	1.00	1.00	1.00	1.00	1.00	0.09	0.10	0.28	0.12	0.07
ADH1	1.00	1.00	1.00	1.00	1.00	1.00	0.09	0.10	0.28	0.12	0.07
ADH4	1.00	1.00	1.00	1.00	1.00	1.00	0.09	0.10	0.28	0.12	0.07
SFA1	1.00	1.00	1.00	1.00	1.00	1.00	0.09	0.10	0.28	0.12	0.07
ADH3	1.00	1.00	1.00	1.00	1.00	1.00	0.09	0.10	0.28	0.12	0.07
ADH5	1.00	1.00	1.00	1.00	1.00	1.00	0.09	0.10	0.28	0.12	0.07
PDC1	0.09	0.09	0.09	0.09	0.09	0.09	1.00	0.79	0.44	0.48	0.30
PDC6	0.10	0.10	0.10	0.10	0.10	0.10	0.79	1.00	0.53	0.53	0.31
PDC5	0.28	0.28	0.28	0.28	0.28	0.28	0.44	0.53	1.00	0.40	0.23
BAT2	0.12	0.12	0.12	0.12	0.12	0.12	0.48	0.53	0.40	1.00	0.28
BAT1	0.07	0.07	0.07	0.07	0.07	0.07	0.30	0.31	0.23	0.28	1.00

Supplementary Table S3. Similarity values among genes in *valine degradation* pathway obtained by method Wang on BP ontology

	ADH3	ADH4	ADH5	ADH2	ADH1	SFA1	PDC1	PDC5	PDC6	BAT1	BAT2
ADH3	1.00	0.92	0.89	0.82	0.88	0.93	0.66	0.63	0.74	0.72	0.74
ADH4	0.92	1.00	0.87	0.85	0.87	0.87	0.67	0.66	0.75	0.74	0.75
ADH5	0.89	0.87	1.00	0.88	0.84	0.80	0.64	0.65	0.75	0.76	0.75
ADH2	0.82	0.85	0.88	1.00	0.92	0.93	0.46	0.66	0.79	0.75	0.79
ADH1	0.88	0.87	0.84	0.92	1.00	0.91	0.62	0.65	0.77	0.74	0.76
SFA1	0.93	0.87	0.80	0.93	0.91	1.00	0.61	0.63	0.75	0.57	0.74
PDC1	0.66	0.67	0.64	0.46	0.62	0.61	1.00	0.98	0.70	0.66	0.70
PDC5	0.63	0.66	0.65	0.66	0.65	0.63	0.98	1.00	0.73	0.68	0.73
PDC6	0.74	0.75	0.75	0.79	0.77	0.75	0.70	0.73	1.00	0.93	1.00
BAT1	0.72	0.74	0.76	0.75	0.74	0.57	0.66	0.68	0.93	1.00	0.47
BAT2	0.74	0.75	0.75	0.79	0.76	0.74	0.70	0.73	1.00	0.47	1.00

Supplementary Table S4. Similarity values among genes in *valine degradation* pathway obtained by method WIS on BP ontology

	ADH2	ADH1	ADH4	SFA1	ADH3	ADH5	PDC1	PDC6	PDC5	BAT2	BAT1
ADH2	1.00	0.82	0.36	0.35	0.88	0.82	0.21	0.23	0.21	0.08	0.08
ADH1	0.82	1.00	0.36	0.30	0.81	0.88	0.28	0.21	0.28	0.07	0.07
ADH4	0.36	0.36	1.00	0.69	0.45	0.36	0.27	0.26	0.27	0.11	0.11
SFA1	0.35	0.30	0.69	1.00	0.28	0.30	0.24	0.28	0.24	0.09	0.09
ADH3	0.88	0.81	0.45	0.28	1.00	0.81	0.20	0.19	0.20	0.08	0.08
ADH5	0.82	0.88	0.36	0.30	0.81	1.00	0.28	0.21	0.28	0.07	0.07
PDC1	0.21	0.28	0.27	0.24	0.20	0.28	1.00	0.84	0.90	0.12	0.12
PDC6	0.23	0.21	0.26	0.28	0.19	0.21	0.84	1.00	0.84	0.13	0.13
PDC5	0.21	0.28	0.27	0.24	0.20	0.28	0.90	0.84	1.00	0.12	0.12
BAT2	0.08	0.07	0.11	0.09	0.08	0.07	0.12	0.13	0.12	1.00	0.92
BAT1	0.08	0.07	0.11	0.09	0.08	0.07	0.12	0.13	0.12	0.92	1.00

Supplementary Table S5, Figure S2 and Figure S3— Correlation with CESSM dataset

We accumulate the results of Seq, Pfam and ECC for the seven methods on IEA+ datasets which is shown in **Figure S2**. WIS performs best which is followed by GIC, Teng and UI. Method JC is the worst in all of the methods. The accumulated results on IEA- datasets are listed in **Figure S3**. WIS also shows great advantage comparing with other methods especially for pairwise methods. The results confirm that WIS can serve as a competitive method to estimate the functional similarity. The groupwise methods perform better in this datasets.

Supplementary Table S5. The accumulated results of Seq, Pfam and ECC for each method. The best results are in bold.

Type	simGIC	simUI	Resnik	Lin	JC	Teng	WIS
IEA+	5.2145	4.9994	5.0678	4.7498	3.9602	5.0304	**5.2467**
IEA-	4.9523	4.7111	4.7937	4.4973	3.795	4.8996	**5.0149**

Supplementary Figure S2. The accumulated result of Seq, Pfam and ECC for each method on IEA+ dataset.

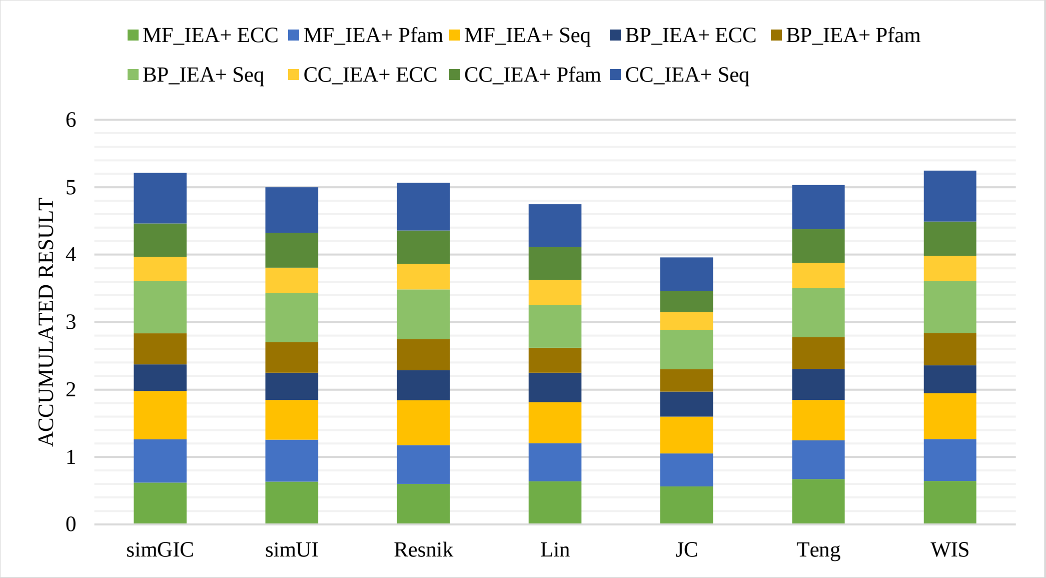

Supplementary Figure S3. The accumulated results of Seq, Pfam and ECC for each method on IEA- dataset.

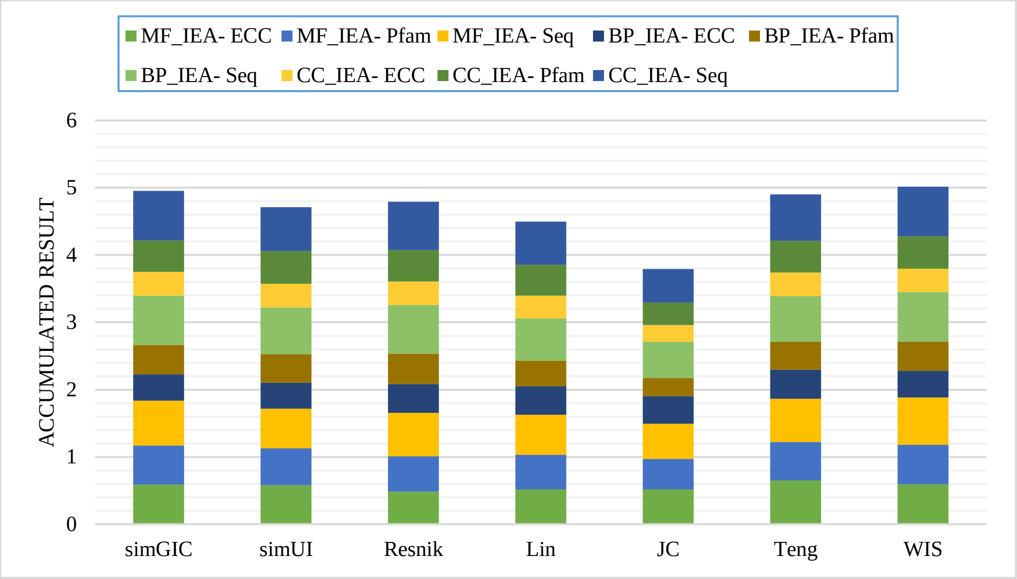

Supplement: Additional file 1: — In the additional file, Figure S1. depecits the distributions of term IC based on CC and MF ontology for each IC calculation model. Tables S1-S4. show the functional similarity values between seleted genes for each method on the functional classification of genes in a biological pathway experiment. Table S5, Figure S2. and Figure S3. list out the accumulated results about correlations on ECC, SeqSim and Res for each method with IEA+ and IEA- on CESSM datasets. The more detail descriptions about the experimental results are shown in the Additional file 1. (PDF 448 kb) [file 12918_2016_359_MOESM1_ESM.pdf]
